# Prognostic gene expression profile testing to inform use of adjuvant therapy: A survey of melanoma experts

**DOI:** 10.1002/cam4.6819

**Published:** 2023-12-14

**Authors:** Suzanne Fastner, Nathan Shen, Rebecca I. Hartman, Emily Y. Chu, Caroline C. Kim, John M. Kirkwood, Douglas Grossman

**Affiliations:** ^1^ Huntsman Cancer Institute University of Utah Health Sciences Center Salt Lake City Utah USA; ^2^ Department of Dermatology University of Utah Health Sciences Center Salt Lake City Utah USA; ^3^ Department of Dermatology Brigham and Women's Hospital and Harvard Medical School Boston Massachusetts USA; ^4^ Department of Dermatology VA Integrated Service Network (VISN‐1) Jamaica Plain Massachusetts USA; ^5^ Department of Dermatology Perelman School of Medicine at the University of Pennsylvania Philadelphia Pennsylvania USA; ^6^ Department of Dermatology Tufts Medical Center Boston Massachusetts USA; ^7^ Departments of Dermatology University of Pittsburgh, UPMC Hillman Cancer Center Pittsburgh Pennsylvania USA; ^8^ Departments of Medicine University of Pittsburgh, UPMC Hillman Cancer Center Pittsburgh Pennsylvania USA

**Keywords:** adjuvant therapy, clinical trial, gene expression profiling, melanoma

## Abstract

**Objectives:**

To investigate current practices and attitudes regarding use of adjuvant immunotherapy and prognostic gene expression profile (GEP) testing among melanoma medical and surgical oncologists.

**Methods:**

An anonymous RedCap‐based survey was emailed to ~300 melanoma experts.

**Results:**

Respondents generally favored adjuvant immunotherapy over observation (73% for all Stage IIIA, 50% for Stage IIB/IIC) and cited a minimum 10‐year recurrence risk of 11%–20% (48%) or 21%–30% (33%) to justify treatment, but acknowledged that risks of serious adverse events may outweigh potential benefits for some Stage IIB/IIC patients. While GEP test results did not strongly influence decision‐making regarding follow‐up or intervention, most were receptive to randomized trials using GEP testing to identify subsets of Stage IIB/IIC (74%) and Stage IB/IIA (54%) patients who may not or may, respectively, benefit from adjuvant therapy.

**Conclusion:**

Although most respondents do not routinely use GEP testing, many would participate in clinical trials to determine clinical utility.

## Abstract 2

In this study, we investigated current practices and attitudes regarding use of adjuvant immunotherapy and prognostic gene expression profile (GEP) testing among melanoma medical and surgical oncologists. Although most respondents do not routinely use GEP testing, many would participate in clinical trials to determine clinical utility.

## 1 INTRODUCTION

Placebo‐controlled clinical trials have demonstrated adjuvant immunotherapy improves recurrence‐free survival in patients with resected American Joint Committee on Cancer (AJCC) Stage III^1^ and Stage IIB/IIC^2^ melanoma, yet potential benefit may be offset by risk of immune‐related adverse events. For example, in Stage IIB/IIC patients, pembrolizumab versus placebo treatment was associated with reduced recurrence rate (15% vs. 24%) but elevated rate (16% vs. 4%) of Grade 3–4 toxicities.^2^ Compared to AJCC staging, prognostic gene expression profile (GEP) testing of primary melanomas may identify individual patients at lower risk of recurrence who could be spared the cost and toxicity of adjuvant therapy, or identify additional patients at higher risk of recurrence who may benefit from adjuvant therapy.

Prognostic GEP testing has been controversial, given its increasing use despite lack of national guidelines' endorsement or evidence supporting clinical interventions based on test results.^3^ For example, approximately 27,800 DecisionDx‐Melanoma (31‐GEP, Castle Biosciences, Inc.) tests were ordered in the United States in 2022,^4^ which may translate to 15%–20% of invasive melanoma cases being tested. However, routine GEP testing continues not to be recommended by the National Comprehensive Cancer Network outside of a study or clinical trial.^5^ Several studies have demonstrated the prognostic value of 31‐GEP testing in prospectively assembled cohorts.^6^, ^7^ A recent study revealed that non‐randomized Stage I–II patients with high‐risk GEP test results, when subjected to scheduled imaging, had earlier detection of recurrences and lower tumor burden than those without GEP testing or scheduled imaging.^8^ On the other hand, it is not clear whether GEP testing provides greater prognostic accuracy than AJCC staging across all melanoma stages, and the optimal timing of adjuvant therapy for unresectable metastatic disease is unknown. Thus, it remains unclear if and how patients should be selected for adjuvant therapy based on GEP test results. There are no randomized controlled trials to guide use of adjuvant therapy based on GEP results for specific cohorts in melanoma, as have successfully been completed in breast cancer.^9^, ^10^

We conducted a survey directed to melanoma medical and surgical oncologists to assess their practices regarding use of GEP testing and adjuvant therapy, and attitudes toward clinical trials to potentially demonstrate clinical utility of GEP testing in determining whether particular stage‐subsets of patients may be spared (or could benefit from) adjuvant therapy.

## 2 METHODS

This study was classified as Exempt from Review (Category 2) and approved by the University of Utah Institutional Review Board (#166093). The authors developed an 18‐question REDCap^11^‐based survey that attempted to capture information regarding current state of melanoma management and decision‐making processes of melanoma experts regarding the use of GEP testing and adjuvant therapy. The survey also focused on their perceived risk of melanoma relapse and balancing the potential benefits and risks of adjuvant immunotherapy in distinct stage‐subsets of patients. A cover page provided contact information for the principal investigator and Institutional Review Board and stated that completion of the survey constituted consent to participate. A survey link was distributed via email to approximately 300 melanoma experts identified at US academic cancer centers (*n* = 102), and nonoverlapping members of the melanoma committees of the Southwest Oncology Group (SWOG, *n* = 117) and Eastern Cooperative Oncology Group/American College of Radiology Informatics Network (ECOG‐ACRIN, *n* = 94). The survey link was resent with a reminder to complete it 2 weeks later. Given the controversial nature of GEP testing, and to encourage responses, the survey was conducted anonymously so respondents did not provide any identifying information and responses could not be tracked to specific individuals. Due to anonymity of the survey, we could not verify if all emails were received or if some individuals received more than one through multiple email addresses.

## 3 RESULTS

The 78 (25%) survey respondents comprised melanoma experts from diverse specialties, including medical oncologists (72%), surgical oncologists (21%), and other specialties (8%) as shown in Table 1. The majority of these experts were based in an academic setting or cancer center (95%), with a significant proportion (>50%) focusing on melanoma. For those that additionally treated other cancers, the majority also had experience treating Merkel cell (87%), basal cell (83%), and squamous cell carcinomas (87%).

**TABLE 1:** Practice patterns of melanoma experts regarding use of adjuvant therapy and GEP testing.

	Total *n* (%)	Medical oncology *n* (%)	Surgical oncology *n* (%)	Other^a^ *n* (%)
Respondents *n* (%)	78/78 (100)	56 (71.8)	16 (20.5)	6 (7.6)
Practice setting (check all that apply)				
Academic or cancer center	74/78 (94.9)	54/56 (96.4)	15/16 (93.8)	5/6 (83.3)
Hospital‐based	3/78 (3.8)	1/56 (1.79)	1/16 (6.25)	1/6 (16.7)
Government	1/78 (1.3)	0 (0)	0/16 (0)	1/6 (16.7)
Community practice	2/78 (2.6)	2/56 (3.57)	0/16 (0)	0 (0)
Melanoma focus (≥50%)	65/78 (83.3)	50/56 (89.3)	13/16 (81.3)	2/6 (33.3)
How do you manage Stage IIIA patients who do not have contraindications to immunotherapy?				
Generally favor adjuvant therapy over observation for all Stage IIIA	15/74 (20.3)	8/55 (14.6)^b^	4/15 (26.7)^b^	3/4 (75)^b^
Generally favor adjuvant therapy over observation unless nodal deposit <1 mm	39/74 (52.7)	29/55 (52.7)	9/15 (60)	1/4 (25)
Generally favor observation over adjuvant therapy for all Stage IIIA	17/74 (23.0)	16/55 (29.1)	1/15 (6.7)	0/4 (0)
Other^c^	3/74 (4.1)	2/55 (3.6)	1/15 (6.7)	0/4 (0)
How do you manage Stage IIB/IIC patients who do not have contraindications to immunotherapy?				
Generally favor adjuvant therapy over observation	36/72 (50.0)	26/55 (47.3)^b^	9/14 (64.3)^b^	1/3 (33.3)^b^
Generally favor observation over adjuvant therapy	17/72 (23.6)	15/55 (27.3)	1/14 (7.1)	1/3 (33.3)
Other^d^	19/72 (26.4)	14/55 (25.5)	4/14 (28.6)	1/3 (33.3)
Do you order the DecisionDx Melanoma test?^e^	16/78 (20.1)	8/56 (14.3)	8/16 (50)	0/6 (0)
Do GEP results influence your management in Stage IB/IIA patients? (check all that apply)				
No, I disregard the test result	47/77 (61.0)	34/56 (60.7)^b^	10/16 (62.5)	3/5 (60)^b^
Yes, decision to refer to surgical oncology for SLNB	6/77 (7.8)	4/56 (7.1)	1/16 (6.3)	1/5 (20)
Yes, decision to offer adjuvant therapy	2/77 (2.6)	2/56 (3.6)	0/16 (0)	0/5 (0)
Yes, decision to recommend surveillance imaging or frequency of surveillance imaging	15/77 (19.5)	12/56 (21.4)	2/16 (12.5)	1/5 (20)
Yes, to determine recommendation for follow‐up frequency interval	11/77 (14.3)	8/56 (14.3)	3/16 (18.8)	0/5 (0)
Other (please describe)^f^	1/77 (1.3)	1/56 (1.8)	0/16 (0)	0/5 (0)
Do GEP results influence your management in Stage IIB/IIC patients? (check all that apply)				
No, I disregard the test result	53/76 (69.7)	37/55 (67.3)^b^	13/16 (81.3)^b^	3/5 (60)^b^
Yes, decision to refer to surgical oncology for SLNB	4/76 (5.3)	3/55 (5.5)	0/16 (0)	1/5 (20)
Yes, decision to offer adjuvant therapy	10/76 (13.2)	9/55 (16.4)	1/16 (6.3)	0 (0)
Yes, decision to recommend surveillance imaging or frequency of surveillance imaging	9/76 (11.8)	7/55 (12.7)	1/16 (6.3)	1/5 (20)
Yes, to determine recommendation for follow‐up frequency interval	6/76 (7.9)	4/55 (7.3)	2/16 (12.5)	0 (0)
Other (please describe)	0/76 (0)	0/55 (0)	0/16 (0)	0 (0)
Do GEP results influence your management in Stage IIIA patients? Check all that apply				
No, I disregard the test result	55/76 (72.4)	41/55 (74.5)^b^	11/16 (68.8)^b^	3/5 (60)^b^
Yes, decision to refer to surgical oncology for SLNB	1/76 (1.3)	0/55 (0)	0/16 (0)	1/5 (20)
Yes, decision to offer adjuvant therapy	14/76 (18.4)	11/55 (20)	3/16 (18.8)	0/5 (0)
Yes, decision to recommend surveillance imaging or frequency of surveillance imaging	5/76 (6.6)	4/55 (7.3)	1/16 (6.3)	0/5 (0)
Yes, to determine recommendation for follow‐up frequency interval	6/76 (7.9)	4/55 (7.3)	1/16 (6.3)	1/5 (20)
Other	0/76 (0)	0/55 (0)	0/16 (0)	0/5 (0)

^a^ Other includes three dermatologists, one pathologist, one radiation oncologist, and one pediatric oncologist.

^b^ Some respondents declined to answer this question.

^c^ One surgical oncologist generally favors adjuvant therapy over observation for nodal deposit >0.3 mm. One medical oncologist asks patients to make informed decision after discussing known data about adjuvant treatment and the lack of data in IIIA setting. Another medical oncologist favors adjuvant targeted therapy for Stage IIIA if BRAF‐mutant positive.

^d^ Six medical oncologists and two surgical oncologists favor adjuvant therapy in stage IIC but not Stage IIB. Two surgical oncologists, and one dermatologist take an individualized approach factoring in age, comorbidities, patient preferences, tumor pathology, and patient risk tolerance.

^e^ 94.7% of surveyed providers were familiar with the DecisionDx‐Melanoma GEP test.

^f^ One medical oncologist may use the GEP result as a “tie‐breaker” for timing of surveillance imaging or adjuvant therapy, but it would not have any impact on decision for sentinel node biopsy.

The management of Stage IIIA and Stage IIB/IIC melanoma patients without contraindications to immunotherapy varied across specialties and is detailed in Table 1. For stage IIIA patients with no contraindications to immunotherapy, 67% of medical oncologists and 87% of surgical oncologists generally favored adjuvant therapy over observation. For stage IIB/IIC patients with no contraindications to immunotherapy, 47% of medical oncologists and 64% of surgical oncologists favored this approach. Overall, more respondents favored adjuvant therapy over observation (54/74 or 73% of total respondents) for Stage IIIA patients without contraindications to immunotherapy than for Stage IIB/IIC patients (36/72 or 50% of total respondents). For Stage IIIA patients, 15/74 (20%) total respondents favored adjuvant therapy for all patients, 39/74 (53%) total respondents favored adjuvant therapy unless nodal deposit was <1 mm, and 4% cited additional influencing factors (Table 1, footnotes). For Stage IIB/IIC patients, 26% of total respondents cited additional influencing factors. While most (95% of total) respondents were familiar with the 31‐GEP test, GEP test results did not influence most respondents' clinical decision‐making in regard to follow‐up or intervention for Stage IB/IIA (47/77 or 61%), Stage IIB/IIC (57/76 or 70%), or Stage IIIA (55/76 or 72%) patients.

In terms of attitudes toward risk, many respondents felt that a minimum of 11%–20% (31/64 or 48% of total) or 21%–30% (21/64 or 33% of total) 10‐year risk of relapse was sufficient to recommend adjuvant therapy (Table 2). A substantial majority (62/69 or 90% of total) of experts across all specialties agreed that for a subset of Stage IIB/IIC/IIIA patients, risk of serious adverse events outweighs potential benefit of adjuvant immunotherapy. Most (51/69 or 74% of total) agreed that there was equipoise and were comfortable with a clinical trial randomizing low‐risk (by GEP test) Stage IIB/IIC/IIIA melanoma patients to adjuvant therapy versus observation, to examine if such patients could be spared the cost and toxicity of treatment. There was less agreement (38/68 or 56% of total) that for a specific subset of Stage IB/IIA patients, risk of relapse warrants evaluation of potential benefit of adjuvant immunotherapy. Just over half of respondents (37/68 or 54% of total) felt there was equipoise in randomizing high‐risk (by GEP test) Stage IB/IIA melanoma patients to adjuvant therapy versus observation, to potentially show such patients could benefit from treatment.

**TABLE 2:** Attitudes of melanoma experts toward risk thresholds and clinical trials incorporating GEP testing and adjuvant therapy.

	Total *n* (%)	Medical oncologists *n* (%)	Surgical oncologists *n* (%)	Other^a^ *n* (%)
Lowest 10‐year risk of melanoma relapse that would motivate you to recommend adjuvant therapy for a patient with resected melanoma				
5%–10%	5/64 (7.8)	1/48 (2.1)^b^	3/12 (25)^b^	1/4 (25)^b^
11%–20%	31/64 (48.4)	22/48 (45.8)	6/12 (50)	3/4 (75)
21%–30%	21/64 (32.8)	18/48 (37.5)	3/12 (25)	0/4 (0)
31%–40%	2/64 (3.1)	2/48 (4.20)	0/12 (0)	0/4 (0)
>40%	5/64 (7.8)	5/48 (10.4)	0/12 (0)	0/4 (0)
Generally agree that there is a subset of Stage IIB/IIC/IIIA patients in whom the risk of serious adverse events outweighs the potential benefit of adjuvant immunotherapy	62/69 (89.9)	46/52 (88.5)^b^	13/13 (100)^b^	3/4 (75)^b^
Agree there is equipoise in a clinical trial randomizing low‐risk (by GEP test) Stage IIB/IIC/IIIA melanoma patients to adjuvant therapy vs. observation^c^	51/69 (73.9)	39/51 (76.5)^b^	10/14 (71.4)^b^	2/4 (50)^b^
Generally agree that there is a subset of Stage IB/IIA patients in whom the risk of relapse warrants evaluation of the potential benefit of adjuvant immunotherapy	38/68 (55.9)	25/50 (50)^b^	9/14 (64.3)^b^	4/4 (100)^b^
Agree there is equipoise in a clinical trial randomizing high‐risk (by GEP test) Stage IB/IIA melanoma patients to adjuvant therapy vs. observation^d^	37/68 (54.4)	29/50 (58.0)^b^	6/14 (42.9)^b^	2/4 (50)^b^

^a^ Other includes three dermatologists, one pathologist, one radiation oncologist, one pediatric oncologist.

^b^ Some respondents declined to answer this question.

^c^ Five medical oncologists and three surgical oncologists believe there is not equipoise in such a trial as GEP testing is either not useful or has not had enough validation. Two medical oncologists do not believe there is equipoise in such a trial as it would strictly depend on the type of adjuvant therapy.

^d^ Two medical oncologists believe that there is equipoise but would not enroll patients for the following reasons: preferring to rely on patient preferences after knowing clinical data and belief that the risks outweigh the benefits. Similarly, nine oncologists do not believe there is equipoise in such a trial as the risks would outweigh the benefits. Two medical oncologists and five surgeons do not believe that there is equipoise in such a trial and that there is not sufficient data from GEP testing to warrant such a trial. One dermatologist believes there is equipoise in placing Stage IIA patients, but not Stage IB patients in such a trial.

## 4 DISCUSSION

Our survey highlights the complex considerations and differing perspectives regarding use of GEP testing and adjuvant therapy among melanoma experts. Interestingly, we found that a greater number of melanoma medical and surgical oncologists favored adjuvant therapy over observation for all Stage IIIA patients without contraindications to immunotherapy than for Stage IIB/IIC patients (73% vs. 50%), some of whom may have worse prognosis for survival according to the AJCC 8th edition.^12^ This difference may be an indication of lag time between recent FDA approval of adjuvant pembrolizumab for Stage IIB/IIC melanoma based on the Keynote‐716 trial^2^ and its incorporation into current clinical practice.

There are several notable limitations of this survey study. First, we had a low response rate (approximately 25%), although it could not precisely be determined due to the anonymity of its design. While anonymity was intended to encourage candid responses, we could not verify if all emails were received or if some individuals received more than one through multiple email addresses. As we could not characterize the non‐respondents, our results may be influenced by non‐responder bias. Furthermore, as the majority of respondents were medical and surgical oncologists, our findings may not be generalizable to other melanoma specialists such as dermatologists. Finally, we did not ask about other factors that may play a role in GEP testing and adjuvant therapy decision‐making like patient sex, which may be an effect modifier and could be considered as a factor in predicting melanoma prognosis in early‐stage melanoma patients.^13^

The increasing use of the 31‐GEP test^4^ seems at odds with the relatively small number of respondents we found who actually order the test, suggesting it is primarily being ordered by practitioners other than medical and surgical oncologists or in nonacademic settings which were not targeted by our survey. We targeted medical and surgical oncologists in the present survey since we viewed they would be the likely providers making decisions regarding use of adjuvant therapy. It would be an interesting follow‐up study to investigate those who do order the test (e.g., dermatologists) to understand their motivations for ordering and how they are using the test results.

The limited use of the 31‐GEP test for clinical management shown here is in accordance with national guidelines,^5^ and is likely due to perceptions that existing data are inadequate to guide decisions regarding sentinel lymph node biopsy, imaging surveillance, or adjuvant therapy. Although several concerns were expressed regarding the trial concepts (Table 2, footnotes), many melanoma medical and surgical oncologists were receptive to trials randomizing Stage IIB/IIC/IIIA low‐GEP risk or Stage IB/IIA high‐GEP risk patients to placebo versus adjuvant therapy. Such approaches may be needed to determine clinical utility of GEP testing where there is current uncertainty and variation in clinical practice.

## AUTHOR CONTRIBUTIONS

**Suzanne Fastner:** Data curation (lead); methodology (supporting); writing – original draft (supporting); writing – review and editing (supporting). **Nathan Shen:** Writing – original draft (supporting); writing – review and editing (supporting). **Rebecca I. Hartman:** Writing – review and editing (supporting). **Emily Y. Chu:** Writing – review and editing (supporting). **Caroline C. Kim:** Writing – review and editing (supporting). **John M. Kirkwood:** Writing – review and editing (supporting). **Douglas Grossman:** Conceptualization (lead); methodology (lead); project administration (lead); writing – original draft (lead); writing – review and editing (supporting).

## FUNDING INFORMATION

The Department of Dermatology at the University of Utah (D.G), the Huntsman Cancer Foundation (D.G.), the VA Integrated Service Network 1 (R.I.H.) and the Department of Defense (R.I.H.).

## CONFLICT OF INTEREST STATEMENT

The authors have no conflict of interest to declare.

## Data Availability

No data, separate from that presented herein, were generated in this work.
